# Prospective surgical solutions in degenerative spine: spinal simulation for optimal choice of implant and targeted device development

**DOI:** 10.1515/iss-2019-1002

**Published:** 2021-04-07

**Authors:** Monique Salchow-Gille, Bernhard Rieger, Clemens Reinshagen, Marek Molcanyi, Joschka Lemke, Uta Brautferger, Kerim Hakan Sitoci-Ficici, Witold Polanski, Thomas Pinzer, Gabriele Schackert

**Affiliations:** Short Care Clinic, Greifswald, Germany; Klinikum Herford, Spine Surgery, Herford, Germany; Department of Neurosurgery, University Hospital of Dresden, Dresden, Germany; University Comprehensive Spine Center, University Hospital of Dresden, Dresden, Germany; Department of Neurosurgery, Brigham and Women’s Hospital, Harvard Medical School, Boston, MA, USA; Institute of Neurophysiology, Medical Faculty, University of Cologne, Cologne, Germany; Department of Neurosurgery, Research Unit for Experimental Neurotraumatology, Medical University Graz, Graz, Austria; Department of Urology, University Hospital of Rostock, Rostock, Germany

**Keywords:** biokinemetrie, cage, discectomy, functional replacement, fusion, minimally invasive spine surgery, prosthesis, range of motion, simulation

## Abstract

**Objectives:**

The most important goal of surgical treatment for spinal degeneration, in addition to eliminating the underlying pathology, is to preserve the biomechanically relevant structures. If degeneration destroys biomechanics, the single segment must either be surgically stabilized or functionally replaced by prosthetic restoration. This study examines how software-based presurgical simulation affects device selection and device development.

**Methods:**

Based on videofluoroscopic motion recordings and pixel-precise processing of the segmental motion patterns, a software-based surrogate functional model was validated. It characterizes the individual movement of spinal segments relative to corresponding cervical or lumbar spine sections. The single segment-based motion of cervical or lumbar spine of individual patients can be simulated, if size-calibrated functional X-rays of the relevant spine section are available. The software plug-in “biokinemetric triangle” has been then integrated into this software to perform comparative segmental motion analyses before and after treatment in two cervical device studies: the correlation of implant-induced changes in the movement geometry and patient-related outcome was examined to investigate, whether this surrogate model could provide a guideline for implant selection and future implant development.

**Results:**

For its validation in 253 randomly selected patients requiring single-level cervical (n=122) or lumbar (n=131) implant-supported restoration, the biokinemetric triangle provided significant pattern recognition in comparable investigations (p<0.05) and the software detected device-specific changes after implant-treatment (p<0.01). Subsequently, 104 patients, who underwent cervical discectomy, showed a correlation of the neck disability index with implant-specific changes in their segmental movement geometry: the preoperative simulation supported the best choice of surgical implants, since the best outcome resulted from restricting the extent of the movement of adjacent segments influenced by the technical mechanism of the respective device (p<0.05).

**Conclusions:**

The implant restoration resulted in best outcome which modified intersegmental communication in a way that the segments adjacent to the implanted segment undergo less change in their own movement geometry. Based on our software-surrogate, individualized devices could be created that slow down further degeneration of adjacent segments by influencing the intersegmental communication of the motion segments.

## Introduction

Advanced degeneration is often the reason, why surgical decompression of neural structures alone is not sufficient, and implants must be used to manage pain.

Published benefits of minimal access spine technology (MAST) procedures include reduced blood loss, fewer wound infections, less postoperative pain, shorter surgical times and shorter hospital stays [1]. At present, numerous percutaneous minimally invasive systems, endoscopy platforms and navigation systems are available for spinal procedures [1, 2]. Planning algorithms are used to preoperatively plan and further reduce the size of the surgical procedure. However, there are no guidelines for selecting devices and for the development of spinal implants to support minimal invasiveness. Which decision-making tools are currently available to substantiate the surgical indication with regards to the surgical strategy and the implant to be selected?

**Figure 1:**
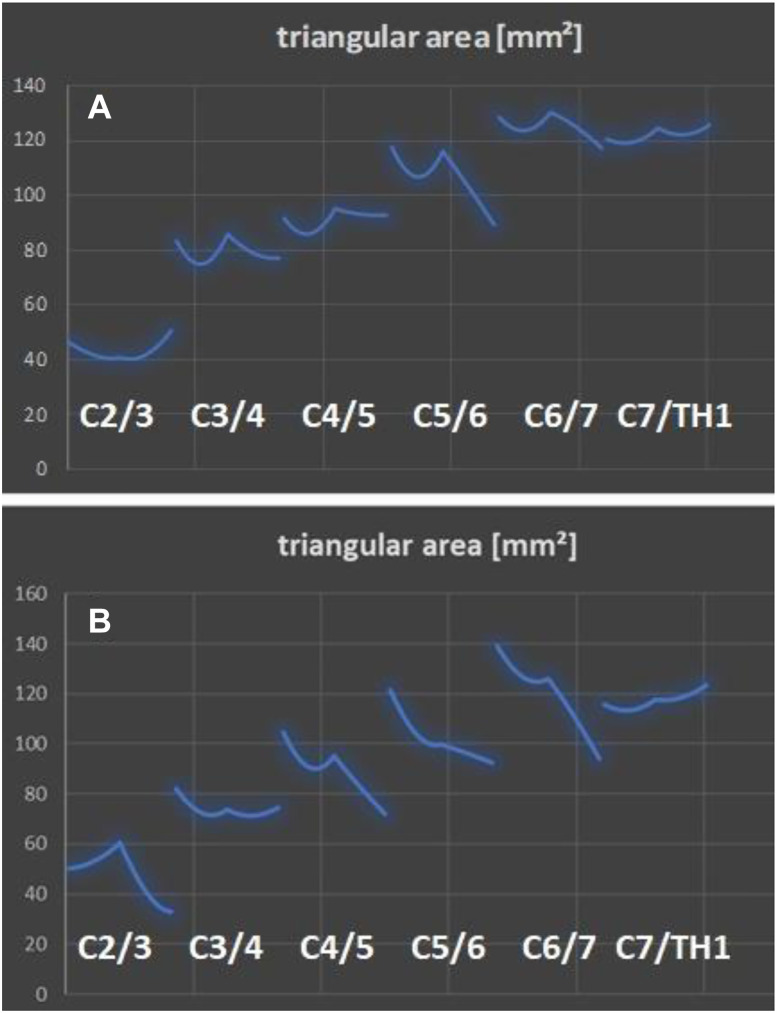
With a range of motion analysis RoM based on the measurements from the biokinemetric triangle (Figure 5) the motion geometry of individual segments and their mutual influence can be studied and algorithmically described. (A) A 33-year-old female patient with segmental degeneration and recent herniated disc C5/6. (B) After six months on conservative therapy and progressive degeneration, communication to the neighboring segments C4/5 and C6/7.

**Figure 2:**
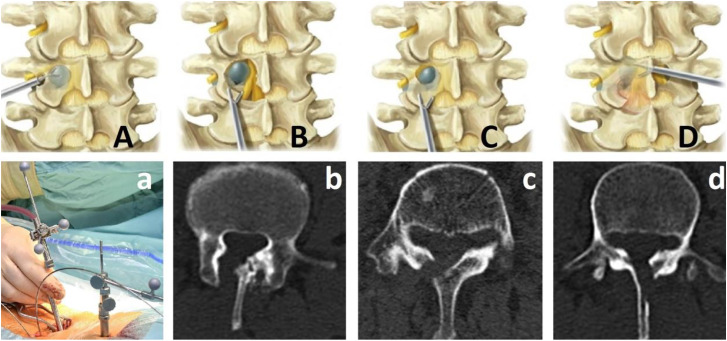
Possible approaches for endoscopy and spondyloscopy (for implant-supported segmental restoration) in the lumbar spine for reduced surgical access modalities: (a) navigation guided endoscopy sleeve for transforaminal approach (A). (b) CT scan after endoscopic interlaminar approach (B). (c) CT scan after spondyloscopic translaminar approach (C). (d) CT scan after spondyloscopic crossover translaminar approach (D).

**Figure 3:**
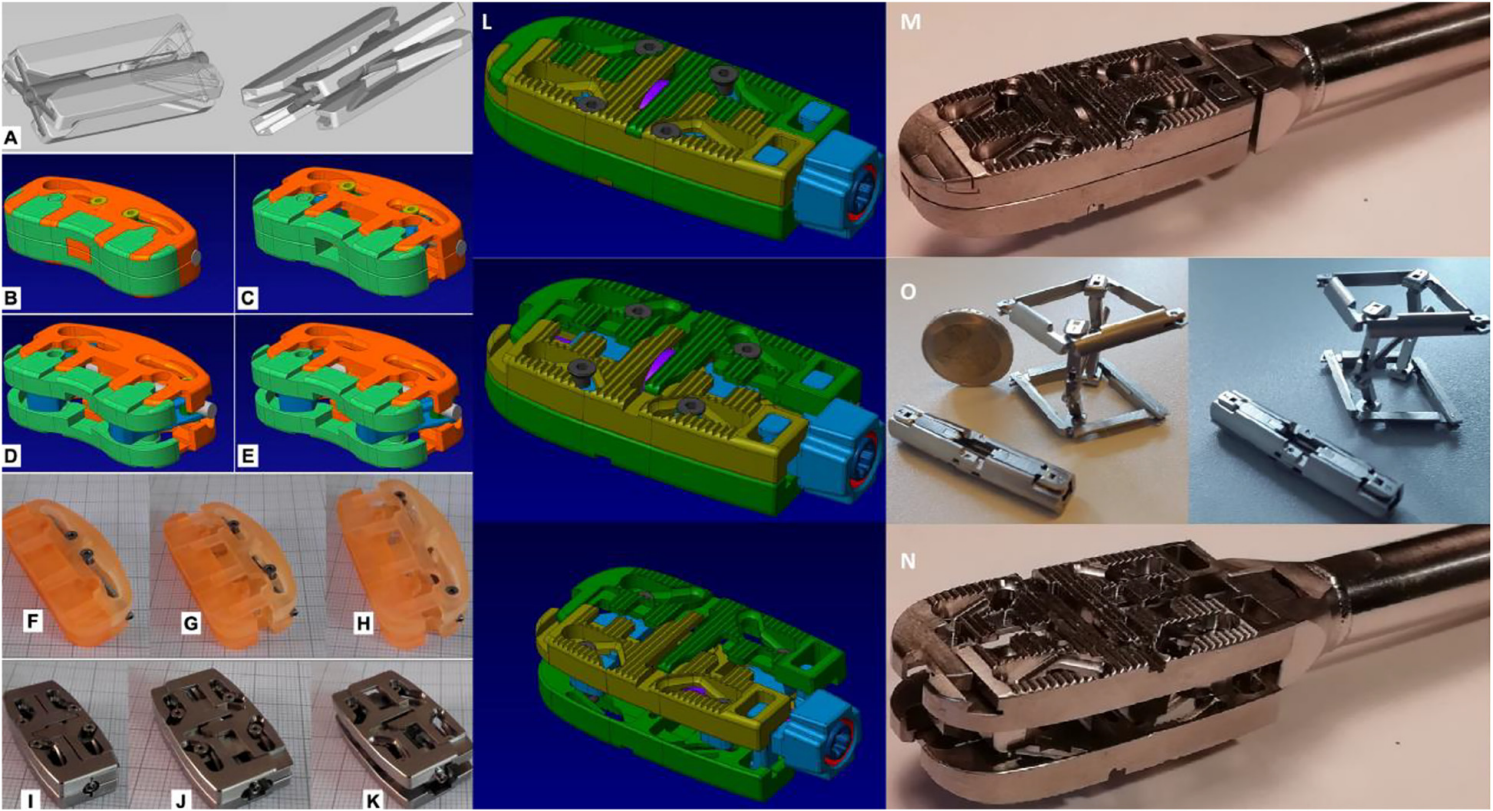
Adjustable devices, adaptable to the needs calculated by the spinal software. (A) First draft of a delta-oblique cage. (B–E) Optimized computer-assisted design (CAD) of delta-oblique expandable cage, featuring closed state (B), horizontal expansion (C), incomplete vertical expansion (D) and complete vertical/horizontal expansion (E). (F–H) First cage prototype in closed (F), horizontally expanded (G) and complete vertical/horizontal expansion (H). (I–K) Further optimized titanium version of the cage using delta-oblique principal when closed (I), horizontally expanded (J) and in complete vertical/horizontal expansion (K). (L) Optimization of the delta technology-based cage for oblique insertion: elyon^TM^. (M, N) Prototyping of the simulation-assisted expandable technique of elyon^TM^. (O) Spondyloscopically implantable preoperatively simulated devices for vertebral body augmentation or replacement.

**Figure 4:**
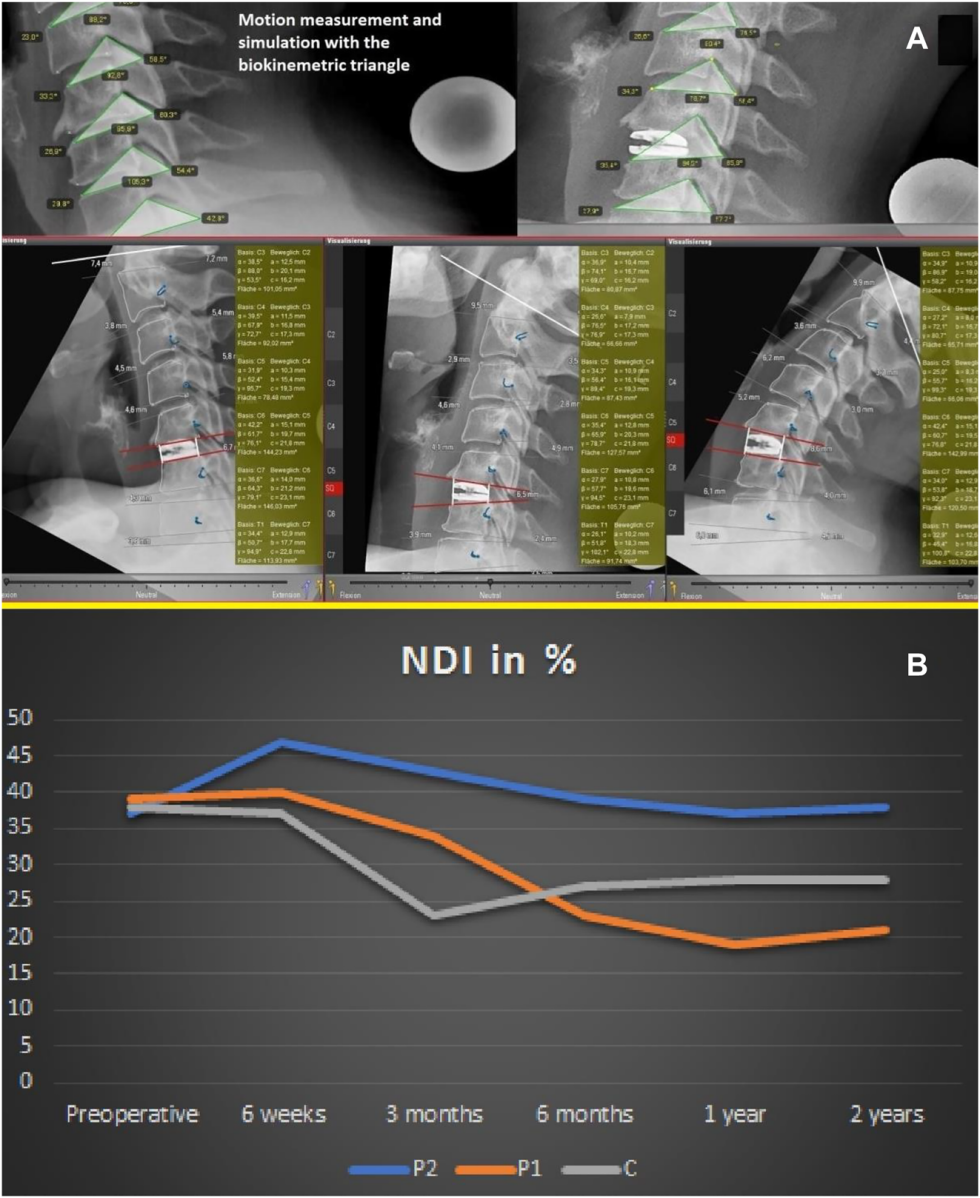
(A) Comparison of pre- and postoperative movement characteristics of a patient enrolled in the PNR Study (NCT02936739: Elastic spine **P**ad [ESP] vs. **R**otaio considering the neck disability index [NDI]) randomized for ESP. (B) Course of the NDI depending on the device/technical design of the implant in patients form the PNS-study and PNR-study: C = Squale^TM^, P1 = Elastic Spine Pad^TM^, P2 = Rotaio^TM^.

Our implant study [3] investigated the correlation between implant-specific altered intersegmental communication and outcome. Modern targeted device development should meet the requirements of minimal invasiveness, and the intersegmental communication should be improved (Figure 1). The difficulties in ultimately achieving optimized patient care that exploits all existing technical aids, lie in the simultaneous clinical implementation of reduced surgical access modalities (Figure 2), the adaptation of the devices (Figure 3), and the outcome-controlled improvement of the underlying biokinemetric surrogate model (Figure 4).

Currently, decisions regarding choice of surgical procedure and implants are primarily based on published study results, the surgeons own experience, and mainly take into account clinical scores and static imaging data. Consequently, in cases of spinal segmental surgery it often remains unclear, whether the surgeon should pursue bony fusion, a prosthetic restauration, a dynamic support or a functional segmental replacement.

**Figure 5:**
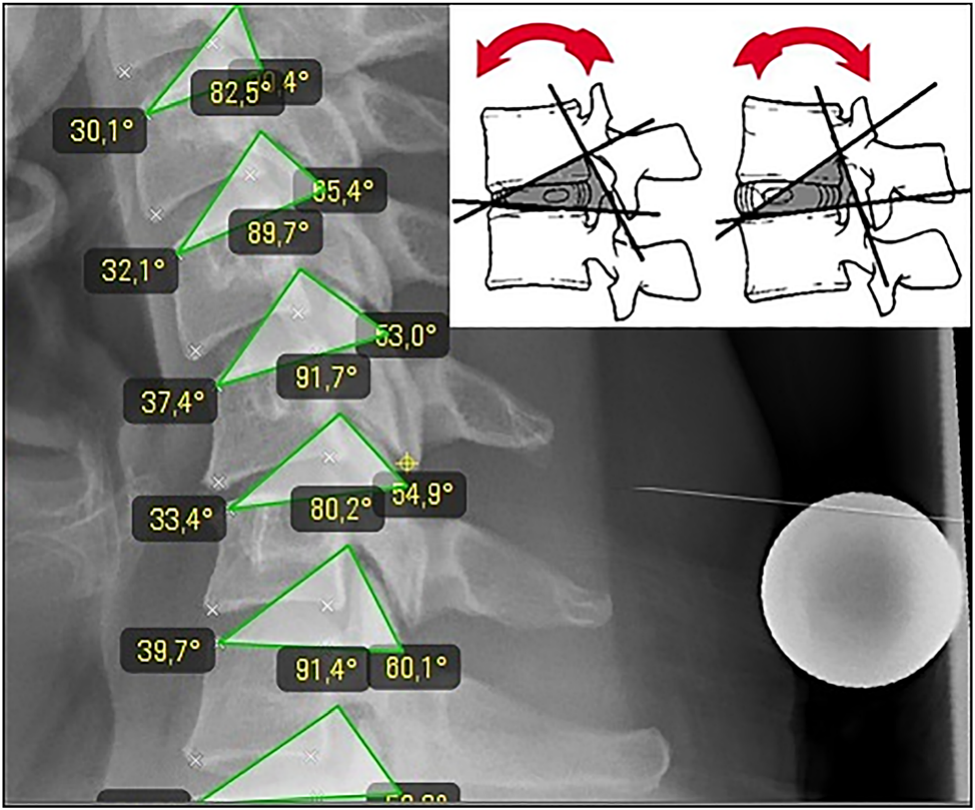
The biokinemetric triangle. The triangle’s baseline is defined by the lower vertebra’s upper plate and reaches from the leading edge of the lower vertebra (1st point) to the ascending lateral facet (2nd point). The 3rd point is defined by the rear edge of the upper vertebra at the roof of the neuroforamen. Because its baseline is fixed, the triangle only changes its height during virtual segmental movement and its triangular area (Figure 1) is proportional to it.

**Figure 6:**
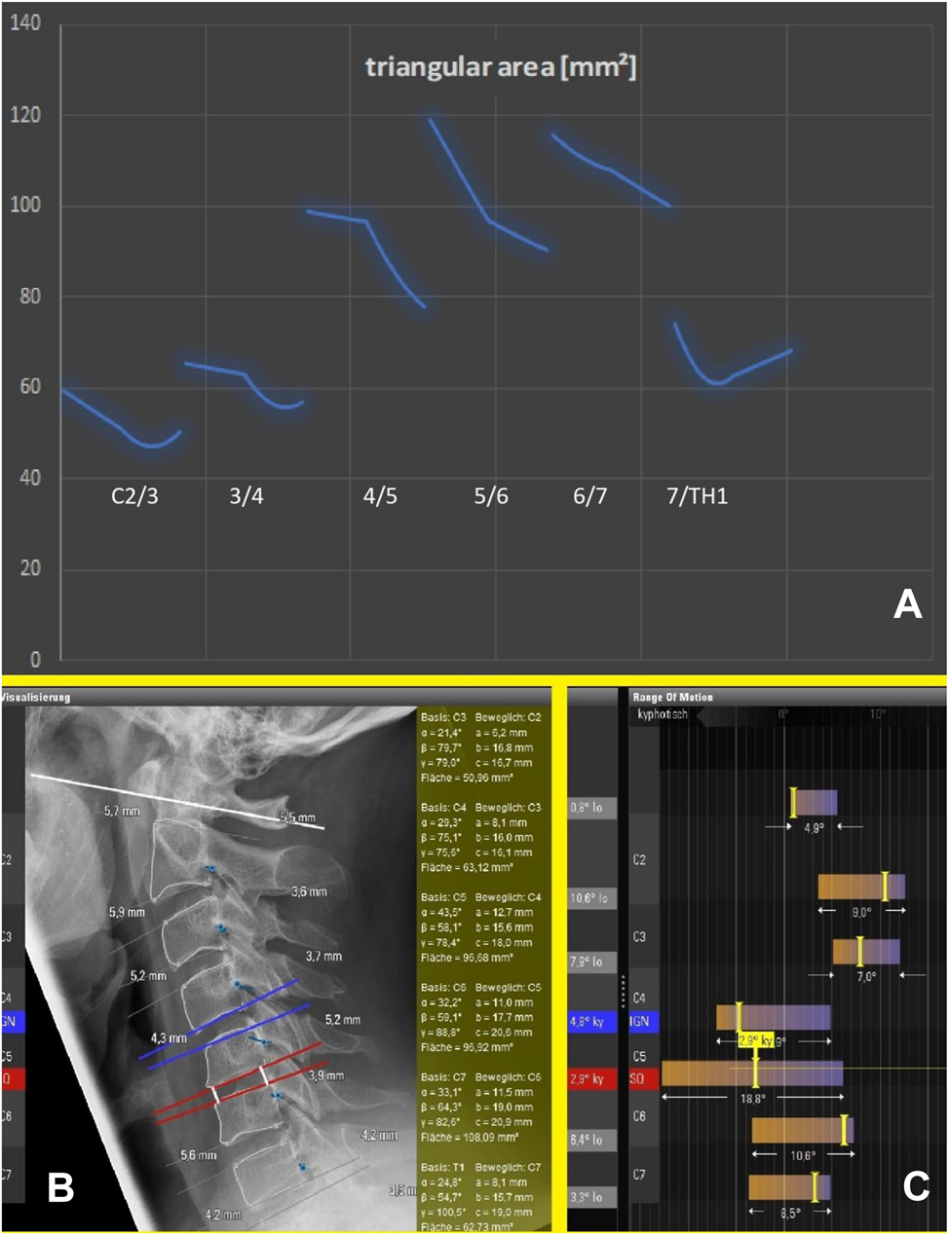
Preoperative motion analysis of a patient enrolled in the PNR Study and indicated for ventral discectomy in C5/C6, who was randomized for Rotaio^TM^: (A) RoM: range of motion analysis by plotting the triangular area data as curves in relation to the percentage of movement of this cervical spine. (B) The movement sequence of the pixel point at the neuroforamen is displayed as a blue curve. The triangle angle data are output in the green diagram. (C) ROM: conventional range of motion analysis based on angle data.

**Figure 7:**
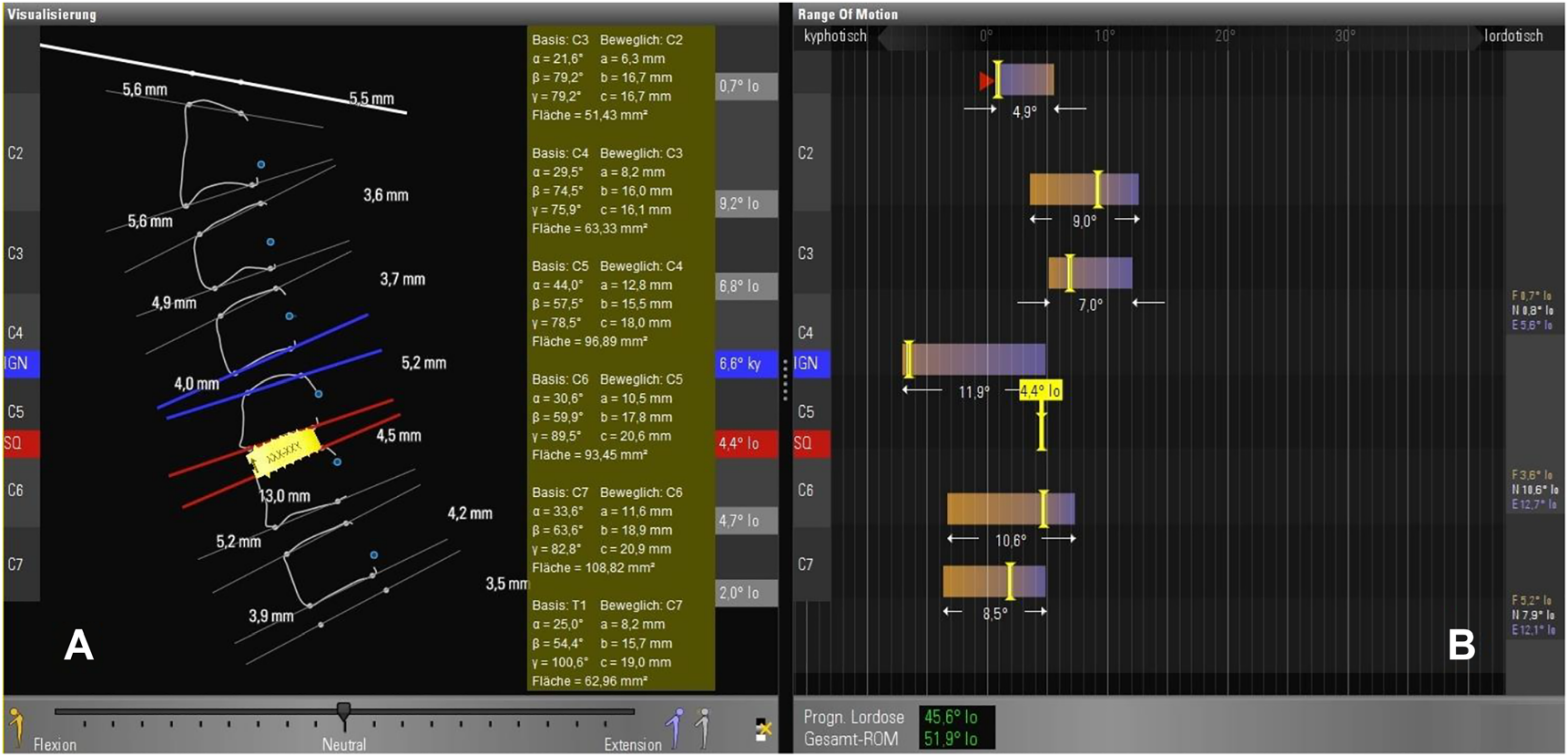
(A) the simulation software determines the optimal device height to 4.5 mm – from 3.9 mm (see Figures 6B–4.5 mm. When enrolling this patient from Figure 6 in the PNR study, the problem arises that prostheses with a height of 4.5 mm are not available and only start with a device height of 5 mm. (B) This ROM analysis is purely virtual and describes the probable movement geometry of adjacent segments if the presented segment C5/C6 is fitted with a cage of height 4.5 mm after ventral discectomy.

Our Prospective Spine^®^ working group has developed a software surrogate and is constantly improving it in an outcome-controlled fashion. The surrogate may aid the surgeon during the surgical decision-making process (Figures 1, 5), since it is able to determine a patient’s optimal segmental height prior to fusion or prosthetic restauration (Figures 6, 7). Furthermore, the software can describe changes in intersegmental communication before and after implant restoration and suggests optimal implant configurations (Figures 6, 7). Apart from aiding the surgeon in choosing patient-optimized implants, the software also allows to compare the real postoperative biokinemetric outcome with the preoperatively simulated outcome with regard to spinal movement characteristics. In consequence, a software-based cut-off analysis is performed, which helps to decide between rigid stabilization and prosthetic restoration, since the segment can be virtually implanted preoperatively, and the resulting movement simulations provide an estimation of how the movement patterns of adjacent levels change depending on the implant used (Figure 7).

The biokinemetric surrogate is the description of the motion geometry of individual segments (no forces and no biomechanics are described) and how the individual motion segments communicate with each other through their own motion geometry, which depends on their functionality (Figure 1).

We hypothesized that such a biokinemetric spinal simulation model may help in the selection of approved spinal implants to choose the implant that leads to the best possible outcome. Furthermore, concerning future implants, biokinemetric motion analysis may facilitate targeted device development with effects on the surgical strategy. To support or disprove these hypotheses a three-phase (I–III) protocol is mandatory (Figure 10).

**Figure 8:**
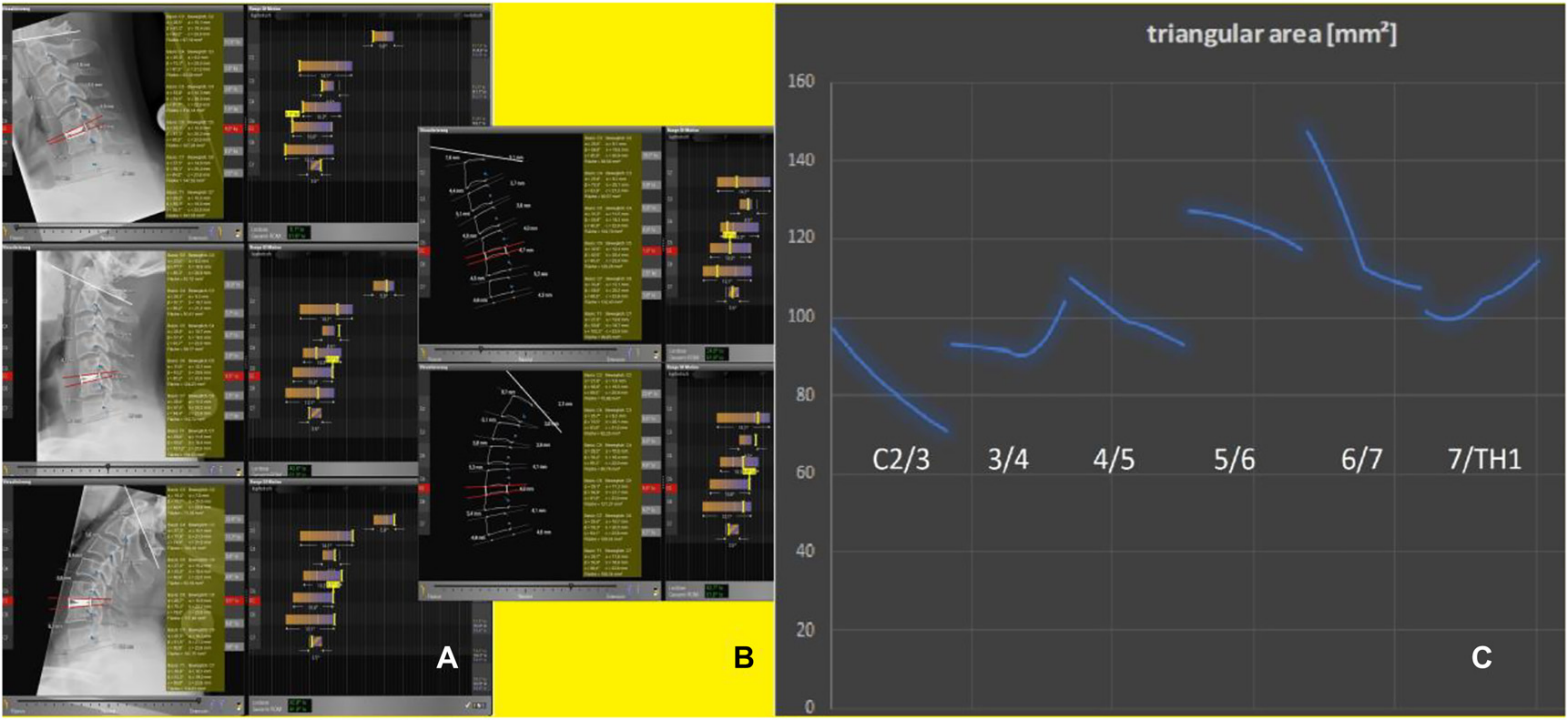
Postoperative motion analysis of the patient from Figure 6. (A) motion recording in inclination, neutral position and reclination, output of triangle data and conventional range of motion analysis (ROM). (B) Display of virtual values based on the surrogate. (C) Postoperative RoM analysis based on the examination with the biokinemetric triangle as a result of real data, data from the surrogate and spline interpolation.

## Materials and methods

**Figure 9:**
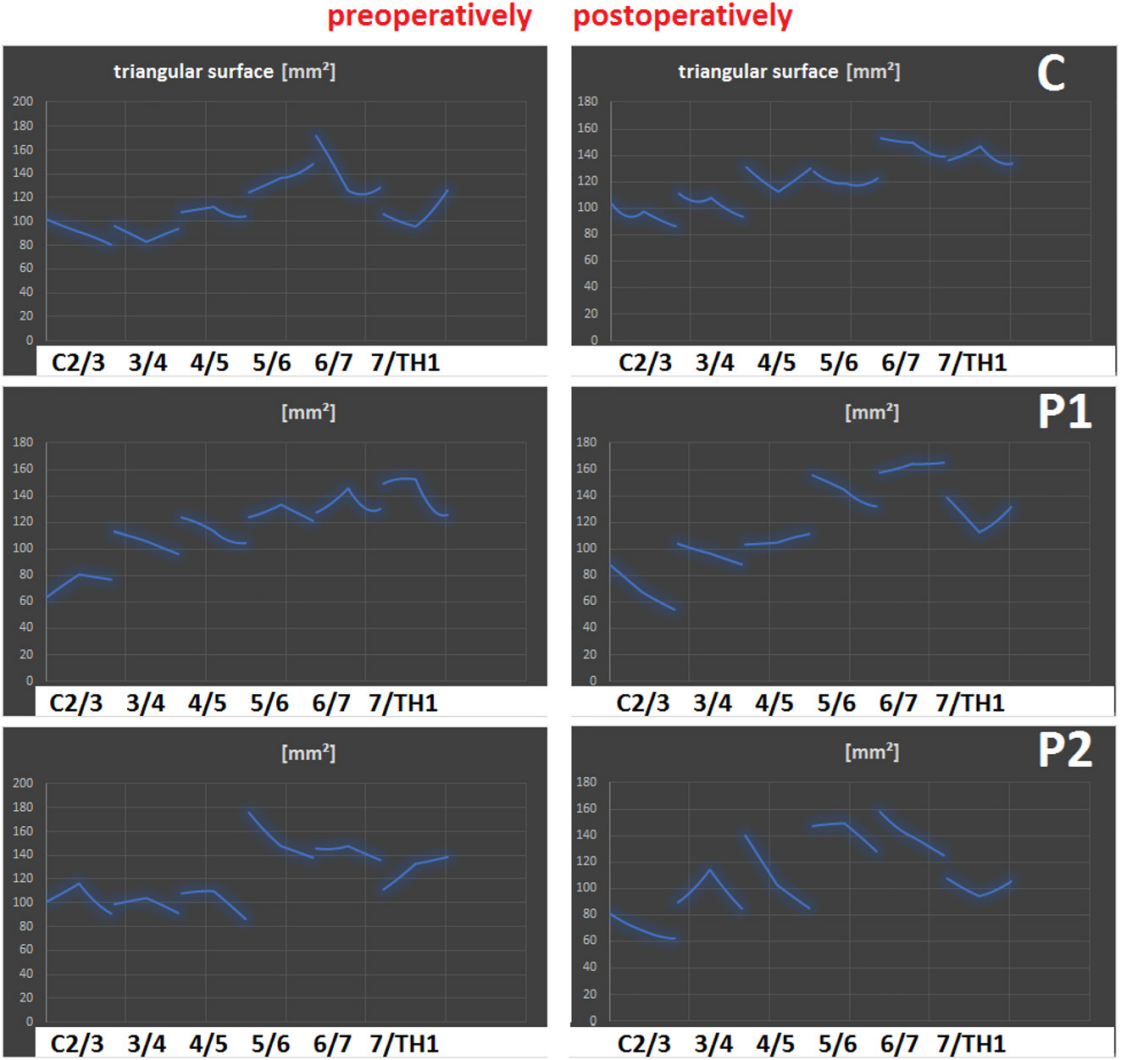
Implantation at C5/C6. Range of motion analysis using the biokinemetric triangle (RoM) after implantation of C (a polyether ketone cage), P1 (a cervical prosthesis which movement mechanism is technically based on an inner and outer core of polycarbonate urethane) or P2 (another prosthesis which movement mechanism is technically based on a sliding hinge joint) at C5/C6. Results from the cervical device studies: PNS and PNR study [3].

- A dynamic simulation model of the spine had to be described and programmed using movement data from real spines: first, standard range-of-motion (ROM) measurements acquired from healthy individuals by videofluoroscopy and described in the literature [e.g. 4], [5], [6], [7], [8], [9], [10], [11], [12], [13], [14] were imported into the software algorithm. This allowed the software to make predictions regarding changes in imaging-based movement geometry of healthy spinal segments, as well as those altered by degeneration. After a patient’s imaging data has been uploaded into the software, the algorithm compares individual movement characteristics with the preinstalled ROM data (Figure 6). This can be used to virtually describe the motion geometry of each segment using radiological motion recordings and thereby detect aberrations due to segmental degenerative changes. Based on the imported ROM data, the software can determine which of the patient’s spinal segments is most pathologically altered (Figures 1, 6). The software then repeats the ROM analysis (Figure 7) by simulating the characteristics of the best segment into the patient’s previously detected worst spinal segment. The worst segment is usually the segment to be implanted, which is indicated by the surgeon, but in most cases it is detected automatically by the software, because the movement geometry of this segment deviates most from the movement geometry stored in the surrogate. This segment is virtually replaced by the best segment of the spine region (**cervical** or **lumbar** spine). The best segment is the segment that has maintained the greatest intersegmental distance and is most closely oriented to the movement geometry of the “healthy” spine stored in the surrogate. Based on this ROM analysis, the software’s algorithm can then calculate how much the intervertebral disc height in the worst segment needs to be surgically altered to improve postoperative sagittal balance. A motion segment or a spine level is defined as two adjacent vertebral bodies connected by an intervertebral disc and the two facet joints. Degeneration usually leads to a reduction in segmental height by decreasing the height of the disc. As a consequence, a resulting subluxation position at the joints can lead to degenerative listhesis. The decrease of the segmental height and listhesis contributes to a sagittal imbalance. For the optimal device selection, the software predicts the optimal configuration of the device (e.g. cage height and size) to counteract this sagittal imbalance and to improve sagittal balance (Figure 7). For stabilizing operations in listhesis, the software simulation also predicts the distance required for improved realignment.
- A measuring parameter suitable to identify implant-specific changes in the movement geometry had to be developed which could be correlated with the outcome: for this simulation software, we created a plug-in based on the “biokinemetric triangle” surrogate parameter to allow biokinemetric calculations [15], [16], [17], [18], [19], [20]. The triangle’s baseline is defined by the lower vertebra’s upper plate and reaches from the leading edge of the lower vertebra (1st point) to the ascending facet (2nd point). The 3rd point is defined by the rear edge of the upper vertebra at the roof of the neuroforamen (Figure 5). Because its baseline is fixed, only the triangle’s height changes during virtual movement. This change in the triangle’s height defines the segmental movement characteristics of the pixel point (Figure 6B) at the roof of the neuroforamen. During movement, the third point is deflected in a characteristic way to prevent movement-related damage to the nerve root. The characteristic curves displayed in the diagrams (Figures 1, 6, 8, 9) result from the change of the triangle’s surface area and the percentage of total movement at this specific point within the performed movement sequence. The curves describe (from left to right) the change of the triangular surface during movement from the inclination to reclination. The decisive advantage of the biokinemetric triangle is that motion characteristics can be compared with each other at different examination times even when the full motion is not performed [21, 22].

Statistical pattern recognition compares the change in the triangular surface area ∆S during the virtual movement of each segment and calculates ∆*S*/*S*_max_. The spinal section to be considered is therefore described by a series of numbers, with each segment represented by a single number. In order to validate the biokinemetric triangle, the functional images required for the individual measurement in inclination, neutral position, and reclination are taken twice at the same time and the resulting simulation results are compared. The Bayes error rate is determined.

The triangle plug-in was introduced in order to screen patients for the risk of adjacent level disease in both **cervical** (Figure 9) and **lumbar** implant surgery (Figure 11), and was developed as part of cervical device studies (Figure 4) carried out in our center [3].

III. After receiving ethical approval (EK 248062016 and EK 249062016), this simulation software had to be used in clinical trials that capture implant-specific outcomes. The use of the software in cervical spine studies was appropriate, since ventral discectomy may be followed by cage-assisted fusion or prosthetic restauration with distinguishable technicality of the prostheses available: in two (❶, ❷) open, prospective, randomized, controlled superiority studies (PNS study: NCT02936765 and PNR study: NCT02936739) the simulation software was established [3]:
❶ **PNS** study: Comparison of software-assisted implantation of Elastic Spine **P**ad^TM^ (P1) with respect to postoperative change in neck disability index (**N**DI) with the conventional disc spacer **S**quale^TM^ (C) after anterior cervical discectomy for cervical disc prolapse.
❷ **PNR** study: Comparison of software-assisted implantation of Elastic Spine **P**ad^TM^ (P1) with respect to postoperative change in **N**DI with the conventional disc prosthesis **R**otaio^TM^ (P2) after anterior cervical discectomy for cervical disc prolapse.

In the PNS study, the software-assisted implantation of the prosthesis (P1) was compared with the implantation of a polyether–ketone–ketone cage (C), while in the PNR-study the two prostheses (P1, P2) of different technical designs were compared with respect to the NDI. Preoperative software-assisted determination of implant height and pre- and postoperative (6 weeks, 3, 6, 12, 24 months) biokinemetric analyses of the individual cervical spine segments were performed using X-ray functional images in inclination, neutral position and reclination with a gauged X-ray ball for real size estimation purpose (Figures 4A, 5, 8). Segmental movement patterns before and after surgery were subjected to biokinemetric triangular analyses (Figure 9). In addition to the NDI outcome measure (Figure 4B, Table 1), other patient-reported outcomes (Table 1) included the core outcome measures index (COMI), numerical rating scales (NRS) for the neck and for the arm, surgical parameters (operation time, blood loss, complications) and the need for analgesics were recorded perioperatively.

**Table 1:** Preliminary patient-related outcomes from the PNR and partly from the PNS study show differences in the course of the neck disability index (NDI) depending on the implant (*P*2, *P*1, *C*) and common improvement of postoperative numeric rating scales (NRS), core outcome measure index (COMI) and Oswestry disability index (ODI) after implantation.

PNR	Preoperative	Six weeks	Three months	Six months	One year	Two years
***P*2**						
NDI	0.37 ± 0.12	0.47 ± 0.17	0.43 ± 0.15	0.38 ± 0.091	0.30 ± 0.12	0.37 ± 0.11
NRS neck	2.7 ± 1.9	4.1 ± 1.5	3.9 ± 2.1	3.7 ± 2.5	3.6 ± 2.5	3.2 ± 2.9
NRS arm	5.2 ± 3.2	0.51 ± 0.43	2.3 ± 1.5	1.7 ± 1.3	1.5 ± 1.0	1.4 ± 1.5
COMI	5.4 ± 2.7	2.1 ± 1.9	1.9 ± 1.5	2.3 ± 1.4	2.5 ± 1.4	3.0 ± 1.1
ODI %	43 ± 22	27 ± 17	32 ± 23	29 ± 12	33 ± 15	35 ± 27
***P*1**						
NDI	0.39 ± 0.17	0.40 ± 0.23	0.34 ± 0.26	0.23 ± 0.12	0.19 ± 0.13	0.21 ± 0.19
NRS neck	3.3 ± 2.1	2.7 ± 2.0	2.8 ± 1.3	1.7 ± 1.5	1.5 ± 1.3	1.5 ± 1.0
NRS arm	6.3 ± 2.7	1.3 ± 1.1	1.5 ± 0.95	1.5 ± 1.1	1.2 ± 1.0	1.9 ± 0.050
COMI	5.1 ± 3.2	2.2 ± 1.2	1.7 ± 1.4	1.9 ± 1.4	1.9 ± 1.7	1.5 ± 1.0
ODI %	39 ± 12	23 ± 17	29 ± 15	17 ± 9.5	20 ± 10	16 ± 13

PNS						
***C***						
NDI	0.38 ± 0.28	0.37 ± 0.22	0.23 ± 0.19	0.27 ± 0.15	0.28 ± 0.12	0.29 ± 0.17
NRS neck	4.2 ± 1.8	3.1 ± 2.0	2.9 ± 1.9	3.1 ± 1.9	3.3 ± 1.5	3.0 ± 1.7
NRS arm	4.7 ± 2.5	0.75 ± 0.45	0.53 ± 0.34	1.1 ± 0.67	0.60 ± 0.45	0.97 ± 0.35
COMI	6.2 ± 2.7	3.7 ± 2.3	3.2 ± 1.9	3.3 ± 2.5	3.2 ± 1.8	2.9 ± 1.9
ODI %	52 ± 27	35 ± 33	32 ± 17	35 ± 22	29 ± 25	32 ± 18

The hypothesis of both studies was that Elastic Spine Pad^TM^ (P1) acts as a prosthetic replacement as well as an elastic cage and has a beneficial effect on postoperative neck pain after ventral discectomy. The simulation software provided an objective measure of the height of each implant, eliminating putative bias that would affect the main variable NDI, since implants that are too high or too low in dimension induce postoperative neck pain. Currently, both studies, which started in 2016, have been completed after full recruitment and a two-year observation period. In order to demonstrate the versatility of the simulation software for implant selection and development, this article in part presents preliminary results (Figure 4B, Table 1) from both studies to establish the relationship between implant-specific changes in motion geometry and outcome.

### Statistical analysis

When the examination was based on a comparative study, we used SPSS version 23.0 (IBM Corporation, Armonk, NY, USA) for statistical analysis. The outcome was compared using an independent t-test. Demographic data was evaluated using Pearson’s chi-square test. A p<0.05 was considered statistically significant. The error rate of pattern recognition for reproducibility of the measurement was derived from the Bayes decision rule.

The present studies are conducted according to the internationally recognized Good Clinical Practice Guidelines as well as the Declaration of Helsinki.

## Results

### Validation of the software surrogate (**I**, **II**)

Following the approval of the simulation software for medical use, 131 patients (73 women, 58 men) underwent preoperative simulation prior to minimally invasive **lumbar** interbody fusion to determine the required cage height. The mean age was 67.8 years (range, 45–87 years). Prior to simulation, all patients had received functional X-rays to estimate segmental instability. After informed consent existing CT-scans were uploaded together with these functional X-rays to the software in order to perform size calibrations.

Two preoperative simulations, which were closely related in time, could thus be created. The triangular data resulting from both investigations were compared on a data level by comparing the series of numbers resulting from ∆*S*/*S*_max_ of the segments describing the respective spine section. The results of the triangular examination are reproducible after pattern recognition (p<0.05) and change, if the movement-specific functional configuration of the spine is altered (e.g. increased spinal degeneration (Figure 1) or after surgery with device implantation (Figure 9). The same results (p<0.05) were obtained in patients requiring a **cervical** implant after ventral discectomy: n=122; male/female=68/54; mean age=54.7 years (range 27–73 years). After single-level implantation the biokinemetric triangle measurement detected specific changes in the motion characteristics (cervical spine n=122, p<0.01; lumbar spine n=131, p<0.01) in these validation cases.

### Software-triggered development of functional replacement strategy based on data from the PNS Study NCT02936765 and the PNR Study NCT02936739 (**III**)

The reproducibility of the measurements was further examined in patients [n=104; male/female=56/48; mean age=49.7 years (range 23–65 years)] enrolled in the **cervical** device studies (i.e. PNS-study and PNR-study) at different examination times. Significant device-specific changes in the movement characteristics were observed (C: p<0.01, P2: p<0.01, P1: p<0.05) (Figure 9). Within the first three months after surgery the largest decrease in NDI was observed in the cage-treated group (p<0.05). However, after six months, the NDI (C) had increased again, so that ∆ NDI (C) was less than ∆ NDI (P1) (p<0.01). Patients treated with P2 showed a significant increase in the postoperative NDI compared with the preoperative value (p<0.05).

Using the biokinemetric triangle analysis (Figures 6, 7, 8, 9), the largest postoperative change in range of motion (∆RoM=|change of area content from the biokinemetric triangle|) of adjacent levels was observed in the P2-treated patient group: ∆RoM (P2)>∆RoM (C) (p<0.01) and ∆RoM (P1)<RoM (C) (p<0.05). There is a negative correlation between the ∆RoM of adjacent levels and postoperative relief of neck pain. The cervical device studies show that the movement characteristics of the neighboring segments are influenced by the respective implant (Figure 4B). The outcome deteriorates if the implant causes large changes in the ∆RoM of adjacent segments. A similar reduction ∆RoM of neighboring segments needs to be achieved, when transferring these results to facilitate the development of lumbar segmental functional replacement strategy: the sagittal (Figure 11A–C) and coronary (Figure 11D–F) biokinemetric triangles of individual patients are graphically conjugated to simulate the movement geometry of the segments in two planes. The optimal device height is calculated, and the segmental motion curve (Figure 12) is virtually improved by reducing ΔRoM of the neighboring segments. Important manufacturing data to create the artificial weight bearing area for an individual facet joint replacement (Figure 13) is generated after matching this simulation with CT data [21]. This weight-bearing area (Figure 13A) builds a joint surface for an artificial facet joint (lock-move). Navigation-guided implantation of the resultant lock-move device into the interspinous space using cortical bone trajectories provides an artificial facet support (Figure 13B, C).

**Figure 10:**
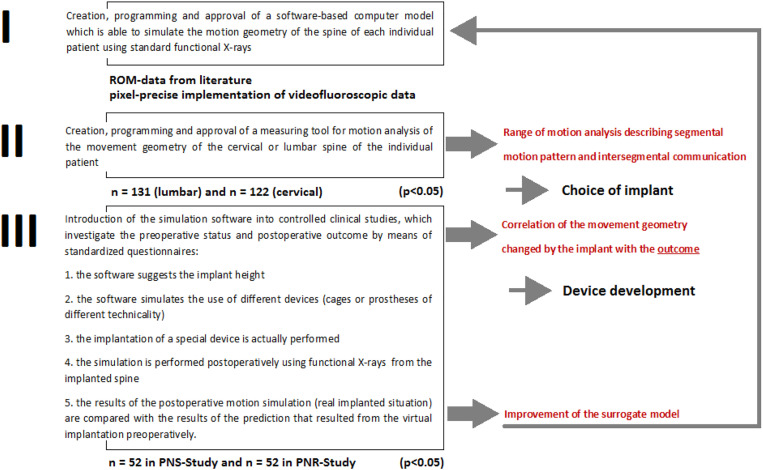
Clinical trial schedule.

**Figure 11:**
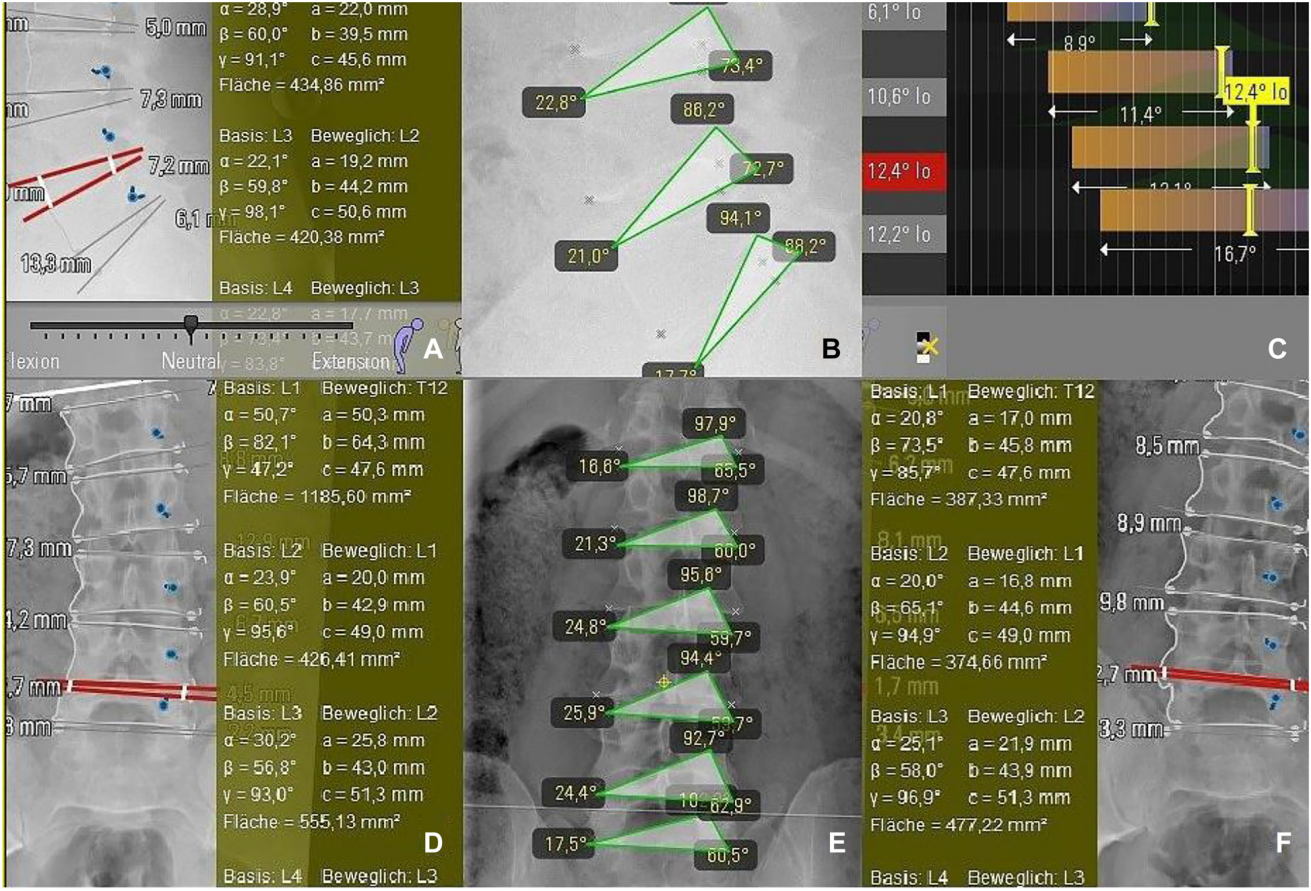
Biokinemetric triangle software plug-in (B, E): simulated software-assisted video fluoroscopic range of motion (ROM) analysis (C) of a healthy male. The virtual movement of the surrogate model is based on plane radiographs acquired in flexion, neutral position (A), extension, oblique left (D), anterior–posterior (E) and oblique right (F). Throughout surgical planning, the position and range of motion of all lumbar segments are continuously displayed by the software. Additionally, the software considers the biokinemetric triangle surrogate parameter, which is used to indirectly quantify the specific movement characteristics (RoM) of every segment (B, E). The triangle’s baseline is defined by the lower vertebra’s upper plate and reaches from the leading edge of the lower vertebra (1st point) to the ascending lateral facet (2nd point). The third point is defined by the rear edge of the upper vertebra at the roof of the neuroforamen in sagittal view. Two possible triangles in coronary view follow the same definition. In the case of a clear definition of image points, any number of triangles which characterize the motion are, of course, conceivable. Because its baseline is fixed, the triangles change only in height during movement. The change in the triangle’s height defines the segmental movement characteristics at the point of the roof of the neuroforamen. During movement, the third point is deflected in a characteristic way, in order to prevent damage to the nerve root.

**Figure 12:**
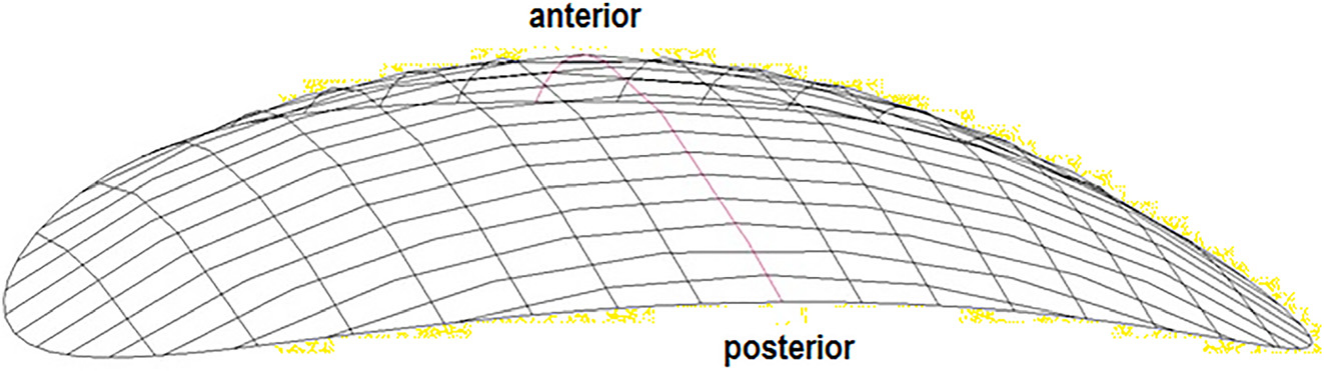
The relative motion of two vertebral bodies from the surrogate expressed as a surface.

**Figure 13:**
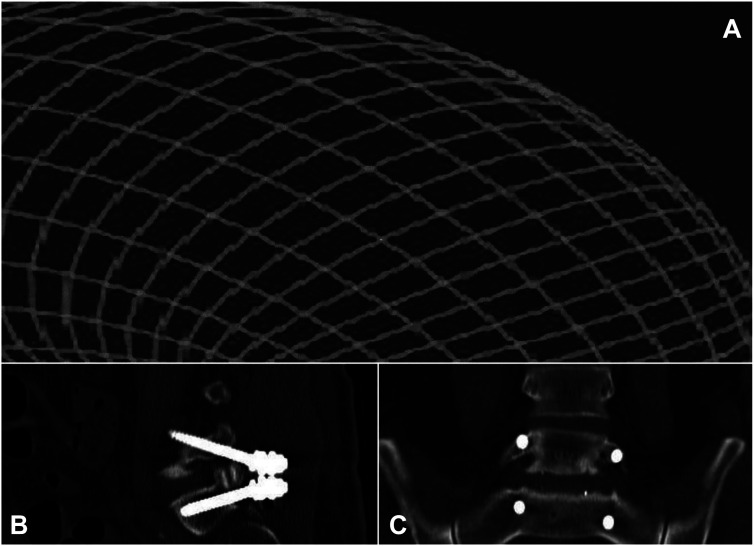
Model of a hypothetical surface for the lock-move [21] (monarticular facet joint support) resulting from the conjugation of the sagittal and coronary triangular data: (A) the software surrogate provided information on what the surface of an artificially produced prosthetic support of the segmental movement, lock-move, should look like, based on the hypothesis that a working segmental replacement must mimic the biological movement characteristics and orientate itself towards improving the sagittal and coronary balance to effectively prevent degeneration. (B) Cortical bone trajectories for the lock-move: CT scan sagittal view. (C) CT scan coronary view: four-year follow-up of a 38-year-old male patient who had undergone surgery 10 times for a prolapse at L5/S1, resulting in damage to the facet joints. He refused rigid stabilization. Supported by spinal simulation software, he was given a polyaxial-head facet joint support with technical conjugation of two circular motions mimicking restricted translation and a monobloc prosthesis at L5/S1 for individualized functional segmental replacement.

**Figure 14:**
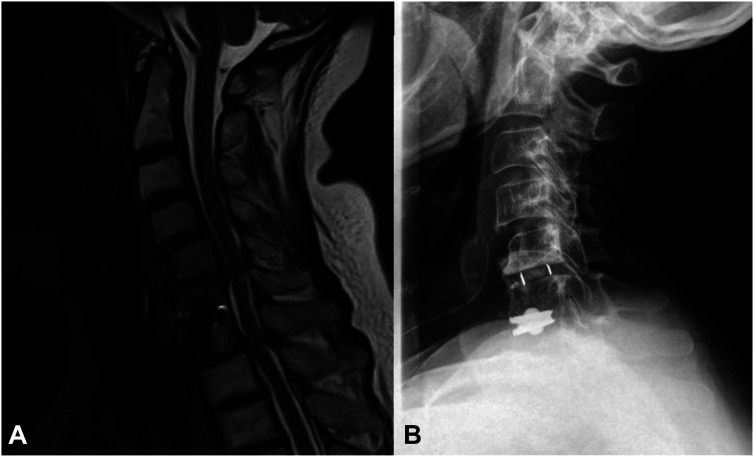
A 52-year-old female patient, who was treated seven years ago with a disc prosthesis based on the technical design “ball and socket” in C6/C7. The reason for a prosthetic restauration is to protect the adjacent segments. (A) MRI scan of the cervical spine: the adjacent segments C5/C6 and C7/Th1 are subject to considerable degenerative changes. (B) Due to myelopathy and cervical stenosis, C5/6 is implanted with a cage. C7/Th1 also requires surgical treatment.

## Discussion

The mathematical description of the movement curves and movement surfaces shows that the motion geometry of the **cervical** spine and **lumbar** spine do not differ in essence so that the biokinemetric results of the cervical device studies can be transferred to the lumbar spine for implant development. If the relative movement of two vertebral bodies in the cervical or lumbar spine from the surrogate is depicted as a surface-area (Figure 12), the resulting function can be mathematically matched with a recurring base and the movement initiation of adjacent segments occurs at the strongest curvature of these surface-areas. It is therefore understandable that the first disc prostheses, which were ball and socket joints, put considerable strain [23] on the adjacent segments, since a ball always has the same curvature, and the motion of the adjacent segments is initiated at any position of the prosthesis (Figure 14). However, even though the cervical and lumbar spine share similar biokinemetric properties, they differ in their biomechanics and the forces acting on them, so that in the lumbar spine the vertebral joints and the intervertebral disc will have to be replaced [21], if the movement segment is to be replaced surgically (Figure 13).

The results from the two cervical device studies teach us to choose the implant, which calms the adjacent segments. Since the simulation allows us to estimate the impact of an already approved implant on adjacent segments, once we have examined this implant pre- and postoperatively, the surrogate helps us to select the device least influencing the adjacent segments. The better the movement geometry of the spine can be modeled, the easier it will be to develop implants for a better outcome after surgical treatment of spinal degeneration. Finally, the surface configuration following individual functional segmental replacement can be calculated based on the knowledge of intersegmental communication.

Using the biokinemetric triangle software plug-in [21, 22, 24], we could study the communication of adjacent segments during movement. Segmental motion patterns show that all segments demonstrate the same motion characteristics, but each individual segment is at a different point within its individual movement sequence at any position of the spinal column. During stabilizing operations, whether rigid or prosthetic, a fracture-like segmental release prior to instrumentation is currently the standard of care. However, as in cases of natural fracture, this leads to irregular movement patterns, which in turn result in callus formation and ultimately fusion of the implanted segment (Figure 14). To create a biokinemetrically functional segmental replacement, the segment’s physiologic range of motion must be determined preoperatively, followed by a simulation of postsurgical conditions with adjustment to adjacent level ROM, to prevent nonphysiological segmental movement and undesired postoperative ossification [23]. Simulation software will be vital in achieving this goal [25], since it allows pre- and postoperative analysis of device-specific changes in segmental motion characteristics for both rigid and prosthetic procedures (Figure 9). Along with outcome analysis, simulation software will fuel the evidence-based optimization of segmental surgery. The optimal prosthetic device will likely have only minimal effects on the movement characteristics of adjacent levels or even support the physiologic ROM of the adjacent segments.

It is important to note that the presented software analysis is based on a motion model of the spine. The individual spine is described by the movement modality of its individual movement segments and their sequence. The motion analysis of an individual spine is performed with a measuring instrument on this model. It had to be excluded that measurements with the help of this model are not measurements of the model and results are relevant for patients and do not result from the spline interpolation of missing measurement points (Figure 8). However, since repeated examinations on individual patients can be clearly assigned to individuals like a fingerprint, it can be assumed that the biokinemetric spine model is relevant. If an “optimal” implant height (Figure 7) is determined preoperatively with the aid of biokinemetric analysis, the implant height results because a relatively healthy segment from the movement section under consideration (**cervical** spine or **lumbar** spine) was virtually inserted in the segment to be implanted. Before this virtual implantation an additional comparison with the database is performed concerning the real size ratios, since a distal intervertebral disc is usually larger than a proximal one. This procedure was chosen when programming the software to take the current degenerative state of the spine into account when selecting implants and to prevent overcorrection. However, if the segment height of a degenerately altered segment is increased by an implant, the balanced state of the spine is approached again without knowing the plumb line “sagittal balance”.

Patients with advanced spinal stenosis or spondylolisthesis benefit from surgical treatment [18, 19]. Preoperative simulation and intraoperative navigation promote the progress of minimal invasiveness in surgical treatment [26]. However, no better neurological and functional long-term outcome has yet been proven [27].

The traditional static view of the spine is based on the lessons learned from surgical care of spinal trauma and juvenile scoliosis [20, 28]. The surgical procedures initially developed for these pathologies were only later adopted for the treatment of spinal degeneration. However, spinal degeneration is a different pathology and consequently requires an antidegenerative perspective in its surgical treatment. With the development of our surrogate simulation environment, we emphasize a dynamic view of the human musculoskeletal axis. The implementation of videofluoroscopic results [4], [5], [6], [7], [8], [9], [10], [11], [12], [13], [14], [15] into the software algorithm allows the resulting surrogate model to provide information about the patient’s individual preoperative and projected postoperative spinal movement characteristics (preoperative simulation).

Previously, when we compared two cohorts undergoing minimally invasive hybrid lumbar interbody fusion (n=132), we found a significantly better outcome at the six-month follow-up when the procedure had been simulated preoperatively (p<0.05) [26]. Additionally, preoperative simulation resulted in lower intraoperative radiation exposure of patients and staff because no sample cages had to be used for radiographic cage height determination. Based on these findings and the present results, we believe that the next developmental step in minimal access spine technologies will entail the implantation of expandable devices that are preset to unfold into preoperatively determined (software-assisted) patient-specific dimensions (Figure 3) and prosthetic fittings must mimic segment-specific movement (Figures 12, 13). This will allow further minimization in the size of the surgical access for segmental restorations, since expandable devices can be implanted in their collapsed state [29], [30], [31]. At the same time, however, further reduction of the approach size will require the development of new access instruments to perform in tandem with the expandable implantation devices [32]. With regard to the expansion mechanism, it will be technically easier for manufacturers to produce implants with fixed preoperatively simulated post-expansion heights (Figure 3), rather than devices that have to be fitted intraoperatively. This will not only reduce surgical time, but will further minimize the complexity of implantation and device development, since reduction distances, necessary device dimensions and optimal device technology are known preoperatively.

## Conclusions

In view of the demographic development, effective and cost-efficient surgical procedures are required for the best possible outcome in spinal degeneration. The application of spinal simulation software—based on the virtual description of segmental movement patterns—helps to reduce the size of minimally invasive surgical procedures requiring implants. The outcome is all better when adjacent segments are less affected by the technical design of the implant. A rigid cage, as well as some prosthetic restorations, may have a greater effect on adjacent segments. Segmental degeneration itself causes overstrain of adjacent segments via intersegmental communication.

The state of degeneration ultimately determines the implant technology to be used in the individual case. The virtual description of intersegmental communication allows us to hypothesize that it is possible to slow down the degenerative progress by individualized implant restoration and conscious influence on intersegmental communication.

The surrogate model will therefore be an invaluable tool for diagnostics (identifying the altered segment, cut-off analysis to support the indication), navigation-controlled intraoperative execution of the simulated procedure (calculating the height of the device and the repositioning distances, supporting intraoperative image data matching), postoperative evaluation of the surgical outcome, and patient-individualized device development.
