# Supplementary material for: Prospective surgical solutions in degenerative spine: spinal simulation for optimal choice of implant and targeted device development
Source: Innov Surg Sci. 2021 Apr 7;6(1):11–24. doi: 10.1515/iss-2019-1002 (PMC8668033; doi:10.1515/iss-2019-1002)
Supplement: Supplementary file 1 [file iss-06-20191002-s001.pdf]

## Reviewer Assessment

# B. Rieger et al.: Prospective surgical solutions in degenerative spine: spinal simulation for optimal choice of implant and targeted device development

### Reviewers' Comments to Original Submission

**Reviewer 1: Pohlemann, Tim**

Date received: 13-Jul-2020
Reviewer recommendation: **Return to author for major modifications**
Reviewer overall scoring: **Medium**

Assessment Form scores: 5 = High/Yes; 3 = Medium/Adequate; 1 = Low

| | | | | | |
|---|---|---|---|---|---|
| Is the subject area appropriate for the journal | 5 | | | | |
| Does the title clearly reflect the paper's content? | | 4 | | | |
| Does the abstract clearly reflect the paper's content | | 4 | | | |
| Do the keywords clearly reflect the paper's content? | | | 3 | | |
| Does the introduction present the problem clearly? | | 4 | | | |
| Are the results/ conclusions justified? | | | 3 | | |
| How comprehensive and up-to-date is the subject matter presented? | 5 | | | | |
| How adequate is the data presentation? | | | 3 | | |
| Are units and terminology used correctly? | 5 | | | | |
| Is the number of cases adequate? | | | 3 | | |
| Are the experimental methods/ clinical studies adequate? | | | 3 | | |
| Is the length appropriate in relation to the content? | | | | 2 | |
| Does the reader get new insights from the article? | 5 | | | | |
| Please rate the practical significance. | | 4 | | | |
| Please rate the accuracy of methods. | 5 | | | | |
| Please rate the statistical evaluation and quality control. | 5 | | | | |
| Please rate the appropriateness of the figures and tables. | 5 | | | | |
| Please rate the appropriateness of the references. | 5 | | | | |
| Please evaluate the writing style and use of language. | 5 | | | | |
| Please judge the overall scientific quality of the manuscript. | | | 3 | | |
| Are the methods used worthy of reproduction in greater deal? | Yes | | | | |
| Would you be willing to review a revision of this manuscript? | Yes | | | | |

**Comments to author:** Highly interesting field and a great amount of knowledge and development work in the field of simulation, development of instruments, implants and virtual planning and simulation. However, by the inclusion of unpublished data, either the character of a review article is blurred by the primary study design or the original article is diluted by the huge amount of information given by the review article. As the both ways are interesting, but not really compatible, I would recommend to separate bot goals and combine all the published data into a review article including most of the given information and figures etc.. Add a separate original article only aiming towards the new and unpublished data. This certainly would increase the impact and would make it easier for the reader to focus on the message! Otherwise congratulation for an unbelievable amount of works and structured development in a cutting edge field of surgery!

**Reviewer 2: anonymous**

Date received: 16-May-2020
Reviewer recommendation: **Return to author for major modifications**
Reviewer overall scoring: **Medium**

Assessment Form scores: 5 = High/Yes; 3 = Medium/Adequate; 1 = Low

| | | | | | |
|---|---|---|---|---|---|
| Is the subject area appropriate for the journal | | | | | |
| Does the title clearly reflect the paper's content? | | 4 | | | |
| Does the abstract clearly reflect the paper's content | | | 3 | | |
| Do the keywords clearly reflect the paper's content? | | | 3 | | |
| Does the introduction present the problem clearly? | | | 3 | | |
| Are the results/ conclusions justified? | | | 3 | | |
| How comprehensive and up-to-date is the subject matter presented? | | 4 | | | |
| How adequate is the data presentation? | | | 3 | | |
| Are units and terminology used correctly? | | 4 | | | |
| Is the number of cases adequate? | | 4 | | | |
| Are the experimental methods/ clinical studies adequate? | | | 3 | | |
| Is the length appropriate in relation to the content? | | | | 2 | |
| Does the reader get new insights from the article? | | | 3 | | |
| Please rate the practical significance. | 5 | | | | |
| Please rate the accuracy of methods. | | 4 | | | |
| Please rate the statistical evaluation and quality control. | | 4 | | | |
| Please rate the appropriateness of the figures and tables. | | 4 | | | |
| Please rate the appropriateness of the references. | 5 | | | | |
| Please evaluate the writing style and use of language. | | | | 2 | |
| Please judge the overall scientific quality of the manuscript. | | | 3 | | |
| Are the methods used worthy of reproduction in greater deal? | Yes | | | | |
| Would you be willing to review a revision of this manuscript? | Yes | | | | |

**Comments to author:** The author presents very novel and even revolutionary methods in spine surgery; however, the text is quite hard to read. The combination of an original article and review makes the manuscript non common. I cannot find a real “scientific” hypothesis, experiment, and results. The author keeps changing issues and uses numerous novel terms and names of devices.
I recommend the author to write the manuscript differently, first “only” an original article describing a case series of patients treated by his novel technique, which is quite impressive. Introduction must be much shorter; parts of it should be in the

discussion. The hypothesis must be clearer. And the discussion should discuss the results of the case series and compare it to other methods.
In addition, the author can write a review about the innovation of his technique.
I suppose both case series and Review can be even published in journals with much higher impact

## Reviewers' Comments to 1st Revised Submission

### Reviewer 1: Pohlemann, Tim

Date received: 12-Oct-2020
Reviewer recommendation: **Return to author for major modifications**
Reviewer overall scoring: **Medium**

Assessment Form scores: 5 = High/Yes; 3 = Medium/Adequate; 1 = Low

| | | | | | |
|---|---|---|---|---|---|
| Is the subject area appropriate for the journal | 5 | | | | |
| Does the title clearly reflect the paper's content? | | | 3 | | |
| Does the abstract clearly reflect the paper's content | | 4 | | | |
| Do the keywords clearly reflect the paper's content? | | 4 | | | |
| Does the introduction present the problem clearly? | | | 3 | | |
| Are the results/ conclusions justified? | | | | 2 | |
| How comprehensive and up-to-date is the subject matter presented? | 5 | | | | |
| How adequate is the data presentation? | | | 3 | | |
| Are units and terminology used correctly? | | 4 | | | |
| Is the number of cases adequate? | 5 | | | | |
| Are the experimental methods/ clinical studies adequate? | 5 | | | | |
| Is the length appropriate in relation to the content? | | | 3 | | |
| Does the reader get new insights from the article? | | 4 | | | |
| Please rate the practical significance. | | 4 | | | |
| Please rate the accuracy of methods. | | 4 | | | |
| Please rate the statistical evaluation and quality control. | | 4 | | | |
| Please rate the appropriateness of the figures and tables. | | | 3 | | |
| Please rate the appropriateness of the references. | 5 | | | | |
| Please evaluate the writing style and use of language. | | | | 2 | |
| Please judge the overall scientific quality of the manuscript. | | | 3 | | |
| Are the methods used worthy of reproduction in greater deal? | Yes | | | | |
| Would you be willing to review a revision of this manuscript? | Yes | | | | |

**Comments to author:** The paper gained some clarity, but still Needs Reorganisation before I can consider it Ready for publication. The Topic is important, the results convincing and virtual Simulation is certainly one of the more promising Methods to improve surgical Outcome in the muscukoskeletal System. However the aim of the paper is not clear and the important data combined from previous studies are slurred as the "organization" and aim of the paper is not clear. A hypothesis is given an page 4 L3-9 but the answer to the Underlying Question is only given in the discussion (p8, L10-14) with

no further Analysis and Underlying data in the results section. Therefore a decision has to be much wether an original article will support the hypothesis ba a Sound analyses of Underlying clinical data and most of the technical can be omitted and referred to incitations. The better solution, in my eyes, would be a summary of all the Research over the years in an "state of the art" paper. But this has to be stated clearly, but different Research Questions should be transparent to the Reader and the main results of the multiple studies should be clearly stated, discussed and interpreted. Much clarity could be gained for sharing the fascinating data and results with the surgical community.

**Reviewer 2: Schwake, Michael**

Date received: 11-Nov-2020
Reviewer recommendation: **Accept in present form**
Reviewer overall scoring: **High**

Assessment Form scores: 5 = High/Yes; 3 = Medium/Adequate; 1 = Low

| | | | | | |
|---|---|---|---|---|---|
| Is the subject area appropriate for the journal | 5 | | | | |
| Does the title clearly reflect the paper's content? | 5 | | | | |
| Does the abstract clearly reflect the paper's content | 5 | | | | |
| Do the keywords clearly reflect the paper's content? | 5 | | | | |
| Does the introduction present the problem clearly? | 5 | | | | |
| Are the results/ conclusions justified? | 5 | | | | |
| How comprehensive and up-to-date is the subject matter presented? | 5 | | | | |
| How adequate is the data presentation? | 5 | | | | |
| Are units and terminology used correctly? | 5 | | | | |
| Is the number of cases adequate? | 5 | | | | |
| Are the experimental methods/ clinical studies adequate? | 5 | | | | |
| Is the length appropriate in relation to the content? | 5 | | | | |
| Does the reader get new insights from the article? | 5 | | | | |
| Please rate the practical significance. | | 4 | | | |
| Please rate the accuracy of methods. | 5 | | | | |
| Please rate the statistical evaluation and quality control. | 5 | | | | |
| Please rate the appropriateness of the figures and tables. | 5 | | | | |
| Please rate the appropriateness of the references. | 5 | | | | |
| Please evaluate the writing style and use of language. | 5 | | | | |
| Please judge the overall scientific quality of the manuscript. | | 4 | | | |
| Are the methods used worthy of reproduction in greater deal? | | | | | |
| Would you be willing to review a revision of this manuscript? | Yes | | | | |

**Comments to author:** Very good modification of the previsions manuscript, I congratulate the authors for their innovative work in spine surgery.

**Reviewer 3: anonymous**

Date received: 17-Nov-2020
Reviewer recommendation: **Return to author for major modifications**

Reviewer overall scoring: **Medium**

Assessment Form scores: 5 = High/Yes; 3 = Medium/Adequate; 1 = Low

| | | | | | |
|---|---|---|---|---|---|
| Is the subject area appropriate for the journal | 5 | | | | |
| Does the title clearly reflect the paper's content? | | | | 2 | |
| Does the abstract clearly reflect the paper's content | | | | 2 | |
| Do the keywords clearly reflect the paper's content? | | | | 2 | |
| Does the introduction present the problem clearly? | | | 3 | | |
| Are the results/ conclusions justified? | | | | | 1 |
| How comprehensive and up-to-date is the subject matter presented? | | | 3 | | |
| How adequate is the data presentation? | | | | | 1 |
| Are units and terminology used correctly? | | | 3 | | |
| Is the number of cases adequate? | | | | | 1 |
| Are the experimental methods/ clinical studies adequate? | | | | | 1 |
| Is the length appropriate in relation to the content? | | 4 | | | |
| Does the reader get new insights from the article? | | 4 | | | |
| Please rate the practical significance. | | | | 2 | |
| Please rate the accuracy of methods. | | | | | 1 |
| Please rate the statistical evaluation and quality control. | | | | | 1 |
| Please rate the appropriateness of the figures and tables. | | | | 2 | |
| Please rate the appropriateness of the references. | | 4 | | | |
| Please evaluate the writing style and use of language. | | 4 | | | |
| Please judge the overall scientific quality of the manuscript. | | | 3 | | |
| Are the methods used worthy of reproduction in greater deal? | No | | | | |
| Would you be willing to review a revision of this manuscript? | Yes | | | | |

**Comments to author:** Thank you for the invitation to review this manuscript. The authors present an interesting method to analyze the intervertebral movement from sagittal video fluoroscopy looking at kinemetric triangles.

The manuscript is classified to be an "Original Article". Therefore, the methodological description of the patient's data is not sufficient. Furthermore, the authors describe innovative techniques which are not directly associated with the analysis of the intervertebral movement. For example, the development of adjustable intervertebral cages or the development of surgical approaches. From my point of view the manuscript should not be published in its current form.

The article contains several innovative developments and techniques in spine surgery, which can be presented by a review. The title might be like "Innovative techniques to analyze segmental spinal stability and restore sagittal balance".

Authors could describe the problem of segmental spinal degeneration with instability and kyphosis within the introduction part. The main part of the review can then explain the technique to analyze the intervertebral movement from sagittal video fluoroscopy looking at kinemetric triangles. The technique is not easy to understand, please describe the technique enough in detail and especially also with enough subtitle below the figures explaining the technique. What is the difference between using the method in cervical, thoracic, and lumbar spine?

Then authors could explain minimally invasive surgical procedures and why they are important to stabilize or not destabilize vertebral segments. Finally, also authors own developments of adjustable cages can be presented since these are connected to the software analyzing the kinemetric triangles and restoring spinal alignment.

Some other points:

Page 4 line 38: It is unclear to me, what defines the best segment and what the worst segment.

Page 4 line 42: You cannot tell much about the sagittal balance, if you do not have standing x-rays of the sagittal whole spine and pelvis.

Page 8 line 18: It is unclear to me, how a preoperative video fluoroscopy can be used to plan the implant size. Fluoroscopy is usually not calibrated to the correct size.

## Authors' Response to Reviewer Comments

### Response to reviewer 1

The paper gained some clarity, but still Needs Reorganisation before I can consider it Ready for publication. The Topic is important, the results convincing and virtual Simulation is certainly one of the more promising Methods to improve surgical Outcome in the muscukoskeletal System. However the aim of the paper is not clear and the important data combined from previous studies are slurred as the "organization" and aim of the paper is not clear.

*The biokinemetric surrogate is the description of the motion geometry (no forces and no biomechanics are described) of individual segments and the way in which the individual motion segments communicate with each other through their own motion geometry, which depends on their functionality. A biokinemetric spinal simulation model can help in the selection of approved spinal implants to choose the implant that leads to the best possible outcome. Furthermore, with regard to future implants, biokinemetric motion analysis supports a targeted device development with effects on the surgical strategy. To support or disprove this hypothesis a three-phase protocol in the methodology seemed to be mandatory for ultimately have a tool that makes implant selection easier for the surgeon and can be used for targeted, outcome-oriented implant development with patient-individual devices, which result consequently from the previous consideration.*
*I. A dynamic simulation model of the spine had to be described and programmed using movement data from real spines. This software had to be approved for medical use. Based on the dynamic aspect of spine this software has nothing in common with the exclusively static geometric plan models of spine, simply orientated to the plump-line called "sagittal balance".*
*II. A measuring instrument had to be developed which was suitable to identify implant-specific changes in the movement geometry which could be correlated with the outcome.*
*III. After positive ethics this simulation-software had to be used in clinical trials that capture implant-specific outcomes. The introduction of the software in two cervical spine studies seemed to be appropriate because after ventral discectomy the cage fusion with different cages and the prosthetic restauration with distinguishable technicality of the prostheses are available. Two studies were necessary due to statistical considerations.*

A hypothesis is given an page 4 L3-9 but the answer to the Underlying Question is only given in the discussion (p8, L10-14) with no further Analysis and Underlying data in the results section.

*Thank you very much your remark. We have now addressed this issue and have put more emphasis on our hypothesis and discussed our data more.*

Therefore a decision has to be much wether an original article will support the hypothesis ba a Sound analyses of Underlying clinical data and most of the technical can be omitted and referred to incitations.

*We followed your advice and omitted for the sake of clarity some of the technical details - but in the end we have built the software to for device selection and targeted device development.*

The better solution, in my eyes, would be a summary of all the Research over the years in an "state of the art" paper. But this has to be stated clearly, but different Research Questions should be transparent to the Reader and the main results of the multiple studies should be clearly stated, discussed and interpreted.
Much clarity could be gained for sharing the fascinating data and results with the surgical community.

*Many thanks again for your helpful comments.*

**Response to reviewer 2**

Very good modification of the previsions manuscript, I congratulate the authors for their innovative work in spine surgery.

*Thank you very much.*

**Response to reviewer 3**

Comments to the Author
Thank you for the invitation to review this manuscript.
The authors present an interesting method to analyze the intervertebral movement from sagittal video fluoroscopy looking at kinemetric triangles.

*With the help of videofluoroscopies in the sagittal and a.p. plane and also with the help of other data that provide information about how the spine moves in its segments, a movement model of the spine has been created. Movement triangles can be defined arbitrarily within this model (e.g. also in the a.p. plane Fig. 6d, e, f). The point C of these motion triangles describes a surface area during the movement which characterizes the relative motion of two vertebral bodies against each other during the movement of the segment.*

The manuscript is classified to be an "Original Article". Therefore, the methodological description of the patient's data is not sufficient. Furthermore, the authors describe innovative techniques which are not directly associated with the analysis of the intervertebral movement. For example, the development of adjustable intervertebral cages or the development of surgical approaches.

*The software helps to understand inter-segmental communication and the outcome analysis shows that the better the outcome of the implant restoration, the more the implant calms the adjacent segments in their movement. The software is designed solely to improve surgical implant care in the area of spinal degeneration. The ability to define the height of the expansion and the dimensions of the expandable cage is based on an understanding of inter-segmental communication. This means that the implants can and will be developed in accordance with a rationale. This simplifies the development of expandable devices and directly influences the surgical strategy and access. This is exactly the statement this article wants to make.*

From my point of view the manuscript should not be published in its current form.
The article contains several innovative developments and techniques in spine surgery, which can be presented by a review. The title might be like "Innovative techniques to analyze segmental spinal stability and restore sagittal balance".
Authors could describe the problem of segmental spinal degeneration with instability and kyphosis within the introduction part.
The main part of the review can then explain the technique to analyze the intervertebral movement from sagittal video fluoroscopy looking at kinemetric triangles. The technique is not easy to understand, please describe the technique enough in detail and especially also with enough subtitle below the figures explaining the technique. What is the difference between using the method in cervical, thoracic, and lumbar spine?

*In spinal degeneration, almost exclusively the cervical spine and lumbar spine are treated with implants (fusion or prosthetic restoration). With regard to the basic movement geometry, which can now be described mathematically (e. g. by an envelope function), there is no difference between the cervical spine, thoracic spine and lumbar spine. With the help of the software, the respective relative motion of two vertebral bodies can always be traced back in the form of an envelope function (area surface of motion), which carries the base e within itself.*

Then authors could explain minimally invasive surgical procedures and why they are important to stabilize or not destabilize vertebral segments.

Finally, also authors own developments of adjustable cages can be presented since these are connected to the software analyzing the kinemetric triangles and restoring spinal alignment.

Some other points:
Page 4 line 38: It is unclear to me, what defines the best segment and what the worst segment.

*The worst segment is usually the segment to be implanted, which is indicated by the surgeon - but in most cases it is detected automatically by the software, because the movement geometry of this segment deviates most from the movement geometry stored in the surrogate. This segment is virtually replaced by the best segment of the spine region (spine region: cervical or lumbar spine). The best segment is the segment that has maintained the greatest intersegmental distance and is most closely oriented to the movement geometry of a healthy spine stored in the surrogate.*

Page 4 line 42: You cannot tell much about the sagittal balance, if you do not have standing x-rays of the sagittal whole spine and pelvis.

*Degeneration usually leads to a reduction in segment height (a mobile segment is defined as two adjacent vertebral bodies connected by an intervertebral disc and the two facet joints) by decreasing the height of the disc. As a consequence, a resulting subluxation position at the joints can lead to degenerative listhesis. The decrease of the segment height and listhesis always contribute to a sagittal imbalance. Standing x-rays of the sagittal whole spine and pelvis can be imported into the software. However, standing x-rays of the sagittal whole spine for single segment treatment (implantation) of the cervical spine or lumbar spine are not done. The kyphosis and lordosis angles of balanced healthy spines vary considerably. "Sagittal balance" is classically defined by the introduction of a plumb line into the observation of the upright non-moving spine. This plumb line simply describes the state of the upright position where the least forces are exerted on the spine. As soon as the spine leaves the upright position, the description of the sagittal balance and angles is of no use. But again, sagittal whole spine X-rays can be incorporated into the software-based analysis*

Page 8 line 18: It is unclear to me, how a preoperative video fluoroscopy can be used to plan the implant size. Fluoroscopy is usually not calibrated to the correct size.

*X-ray balls are used to calibrate the real size ratios (see figure 7A). The software also offers the possibility to use CT images (if available) to determine the real size rations. Videofluoroscopies were used to set up the software surrogate. The stored software surrogate is a model of the moving spine. This model is related to the individual patient by providing three motion recordings of the patient, which are calibrated with the help of a x-ray ball. Imagine that a movement matrix is related to the individual patient to simulate the movement of the spine of the individual patient. No more videofluoroscopies are made of the patients enrolled in the studies.*

*Thank you very much for your helpful comments. We have tried to take them up and incorporate them into the article. The main objective of our article is first of all to understand how the software works and how it influences the actual implant restoration and development without boring the reader with algorithms and machine language.*

## Reviewers' Comments to 2nd Revised Submission

### Reviewer 3: anonymous

Date received: 15-Dec-2020
Reviewer recommendation: **Return to author for minor modifications**
Reviewer overall scoring: **High**

Assessment Form scores: 5 = High/Yes; 3 = Medium/Adequate; 1 = Low

| | | | | | |
|---|---|---|---|---|---|
| Is the subject area appropriate for the journal | 5 | | | | |
| Does the title clearly reflect the paper's content? | | | 3 | | |
| Does the abstract clearly reflect the paper's content | | 4 | | | |
| Do the keywords clearly reflect the paper's content? | 5 | | | | |
| Does the introduction present the problem clearly? | 5 | | | | |
| Are the results/ conclusions justified? | | 4 | | | |
| How comprehensive and up-to-date is the subject matter presented? | 5 | | | | |
| How adequate is the data presentation? | | | 3 | | |
| Are units and terminology used correctly? | 5 | | | | |
| Is the number of cases adequate? | 5 | | | | |
| Are the experimental methods/ clinical studies adequate? | | | 3 | | |
| Is the length appropriate in relation to the content? | 5 | | | | |
| Does the reader get new insights from the article? | 5 | | | | |
| Please rate the practical significance. | | 4 | | | |
| Please rate the accuracy of methods. | | 4 | | | |
| Please rate the statistical evaluation and quality control. | | 4 | | | |
| Please rate the appropriateness of the figures and tables. | | 4 | | | |
| Please rate the appropriateness of the references. | | 4 | | | |
| Please evaluate the writing style and use of language. | | 4 | | | |
| Please judge the overall scientific quality of the manuscript. | | 4 | | | |
| Are the methods used worthy of reproduction in greater deal? | | | | | |
| Would you be willing to review a revision of this manuscript? | | | yes | | |

**Comments to author:** Thank you for the revision, the manuscript has a better structure now and is easier to understand.
I have a few more things to adapt:
In the methods part you write that “patient-reported outcomes included the Core Outcome Measures Index (COMI), Numerical Rating Scales (NRS) for the neck and for the arm, results from the EuroQol EQ-5D, quality of life questionnaire, surgical parameters” were analyzed.
I cannot find these data within you results. You only present radiological outcome parameter. Therefore, you should only conclude about radiological outcome.
Please explain your study design (Figure 1) and your patient specific data at the beginning of the methods part.

## Authors’ Response to Reviewer Comments

Date received: 24-Dec-2020

### Response to reviewer 3

Thank you for the revision, the manuscript has a better structure now and is easier to understand.

*We appreciate the reviewers’ insightful comments.*

I have a few more things to adapt:In the methods part you write that “patient-reported outcomes included the Core Outcome Measures Index (COMI), Numerical Rating Scales (NRS) for the neck and for the arm, results from the EuroQol EQ-5D, quality of life questionnaire, surgical parameters” were analyzed. I cannot find these data within you results.

*We have combined preliminary visit data from the Neck Disability Index, Core Outcome Measures Index (COMI), and Numerical Rating Scales (NRS) for the neck and for the arm from the two cervical spine studies into one table. We plotted the changes in mean NDI for the different implants in the figure 4b. the Protheses P2 caused clicking noises in several patients, which significantly affected the quality of life. We do not refer to these data (EuroQol EQ-5D) because they need to be discussed separately.*

You only present radiological outcome parameter. Therefore, you should only conclude about radiological outcome.

*We agree with the reviewer. Therefore, we have removed the statement about outcome form the conclusion.*

Please explain your study design (Figure 1) and your patient specific data at the beginning of the methods part.

*This is a good point. The outline of the protocol (I-III) belongs in the methods section. Consequently, we have inserted this outline in the methods section. Thank you for your helpful comments. Your interest and suggestions have contributed significantly to this article.*

## Comments by the Editor-in-Chief to 3rd Revised Submission

The reviewer comments have been adequately addressed by the author.
